# Machine learning-based analysis of drug resistance mutations in *Mycobacterium tuberculosis*

**DOI:** 10.1371/journal.pone.0352863

**Published:** 2026-07-10

**Authors:** Athira Thankamani, Biji C L, George Priya Doss C

**Affiliations:** 1 Laboratory of Integrative Genomics, Department of Integrative Biology, School of Bio Sciences and Technology, Vellore Institute of Technology, Vellore, Tamil Nadu, India; 2 Department of Analytics, School of Computer Science and Engineering, Vellore Institute of Technology, Vellore, Tamil Nadu, India; Kohat University of Science and Technology, PAKISTAN

## Abstract

Tuberculosis is a deadly airborne disease caused by *Mycobacterium tuberculosis*. Drug-resistant tuberculosis presents significant challenges for treatment and control of the disease. Resistant strains of *Mycobacterium tuberculosis* arise from specific mutations in the bacterium. Identification and characterization of resistance-associated mutations are crucial for effective treatment strategies because the first- and second-line drugs for the disease target distinct genes in the bacterium and serve different purposes. Our study developed a machine learning prediction model to analyze mutations across multiple drug-resistance types. The proposed framework predicts drug-resistance mutations across four drug-resistance types, including Rifampicin Resistance, Isoniazid Resistance, Multidrug Resistance, and Pre-extensively Drug-Resistant tuberculosis. The NIAID-NIH TB portal is a publicly available dataset of tuberculosis patients, including drug-resistance information. Our study analyzed 3,065 cases of drug-resistant TB. Eight supervised ML algorithms were implemented for the study. A Random Forest classifier with 10-fold cross-validation shows higher predictive performance than the other seven algorithms considered for further analysis. Significant drug resistance mutations were identified using SHapley Additive exPlanations feature importance. The World Health Organisation mutation catalogues, considered the gold standard for drug-resistant mutations, were used to evaluate prediction results. Mutations not reported in the WHO catalogues were identified during the post-prediction comparative analysis stage, as they may represent potential resistance-conferring markers warranting further investigation, including structural and functional validation or experimental validation. The mutations are *rpoB-*I480T, *rpoC*-G332R, L527V, *gyrA*-D94V, *KatG*-G99E, A106V, W191R, W328C, T380I, and M420T. The study further checks the stability and pathogenicity of the mutations using computational tools, including I-Mutant 2.0 and PredictSNP. The findings added more clarity and further evidence for the significance of the mutation, based on its contribution to drug resistance.

## 1. Introduction

Tuberculosis (TB), caused by *Mycobacterium tuberculosis* (*M. tb)*, is one of the most lethal airborne infectious diseases. The disease has posed a significant public health threat since ancient times. TB is the second leading cause of mortality among infectious diseases. According to data published in 2024, an estimated 10.7 to 10.8 million individuals developed TB worldwide in 2023, with approximately 1.23 million deaths reported globally [1]. Drug-resistant TB (DR-TB) contributes substantially to treatment challenges and reduced recovery rates. In 2019, before the COVID-19 pandemic, approximately 465,000 new cases of Multi drug resistant or Rifampicin resistant (MDR/RR-TB) were reported [2,3]. WHO has conducted global surveillance of DR-TB since 1994, systematically collecting and analysing drug resistance data for various anti-TB drugs from multiple countries and regions [4]. Over the past 25 years, the WHO has monitored drug-resistant TB in accordance with guidelines outlined in the sixth edition of the WHO’s global guidance for the surveillance of drug-resistant TB, published in April 2021 [5]. Data are obtained from diverse sources, including drug susceptibility testing (DST) using phenotypic or molecular methods, with a primary focus on rifampicin resistance in patients with confirmed pulmonary TB [6]. In regions lacking routine testing capacity, periodic epidemiological surveys are conducted. Drug resistance in TB is classified by the number and type of drugs affected. Resistance to rifampicin, a key first-line drug, typically results from mutations in the *rpoB* gene and is termed monoresistance is Rifampicin resistance TB (RR-TB) [7–9]. Resistance to isoniazid, another first-line drug, is causes due to mutations in the *inhA* and *katG* genes is known as isoniazid resistance (Hr-TB) [10,11]. Multi drug resistant TB (MDR-TB) is defined as bacteria resistant to first-line drugs rifampicin and isoniazid are classified as multidrug-resistant [12,13]. In the presence of MDR-TB, resistance to fluoroquinolones classifies the condition as Pre-extensively drug resistant (pre-XDR) TB; further resistance to second-line injectable agents elevates the category to extensively drug-resistant (XDR) TB [14–16]. RR-TB is primarily caused by mutations in the *rpoB* gene, particularly within the 81-base-pair rifampicin resistant-determining region (RRDR). Rifampicin is considered the most critical first-line drug, and resistance to it poses greater challenges than resistance to other TB drugs [17,18]. The key definitions for the drug resistance associated terms used for TB research and analysis based on most recent WHO guidelines (2021 and 2023) are represented (S1 Table). The use of antimicrobial drugs in treatment frequently leads to the development of drug resistance, mainly due to genetic alterations in bacterial genomes. Mutations in genes targeted by these drugs contribute to antimicrobial resistance (AMR) and the emergence of new resistant strains. Analysing genomic change patterns is essential for identifying resistance types and prescribing drugs. Patients undergoing first-line TB treatment are at increased risk of developing drug resistance. Continued use of first-line drugs after resistance has emerged is ineffective. Phenotypic drug-susceptibility tests (pDSTs) are routinely used to assess drug resistance. However, identifying resistance alone is insufficient; it is also necessary to determine the specific gene and mutation responsible at the genomic level. Previous studies have employed machine-learning classifiers to analyse the resistance, susceptibility, prevalence, and pathogenicity of mutations [19]. The present analysis focuses on drug resistance mutations across various resistance categories. Genotypic drug-susceptibility test (gDST) results for 21 drugs were aggregated from the NIH-TB portal. The National Institute of Allergy and Infectious Diseases (NIAID) and the National Institutes of Health (NIH) TB portal is a globally accessible repository that compiles data from patients with TB across 12 countries. The portal is managed by an international consortium of physicians, radiologists, and microbiologists, and includes comprehensive socioeconomic, geographic, clinical, laboratory, radiological, and genomic data, with an emphasis on drug-resistant TB cases [20,21]. This study aims to identify and evaluate genomic variants associated with drug-resistant TB by analysing mutation profiles across 3,065 isolates using machine learning. A machine learning (ML) method was developed to identify highly prevalent and significant mutations across different drug-resistant categories of TB data, utilizing an ML approach and SHapley Additive exPlanations (SHAP) analysis. The study further investigates the prevalence and predictive relevance of mutations across resistance phenotypes to uncover robust molecular markers for classification and surveillance. Additionally, it evaluates the stability of mutations common to all drug-resistant TB types, which are highly significant. The results are compared with the WHO Global Catalogues of Mutation, a standardized set of mutation information compiled from global TB cases using standardized protocols. A set of mutations not reported in the WHO catalogues was identified [22–24]. These findings characterize the mutation patterns associated with each type of drug-resistant TB and highlight previously uncharacterized mutations within these categories.

## 2. Methodology

### 2.1. Data curation and analysis workflow

**Fig 1:**
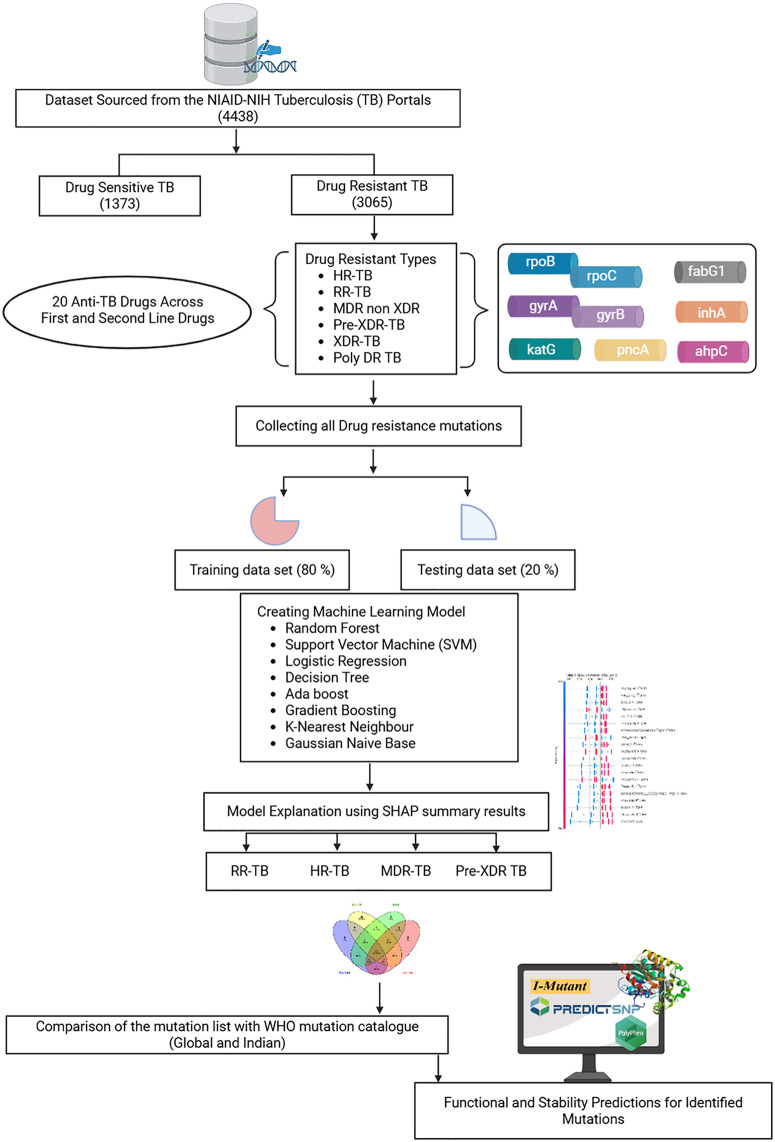
Schematic representation of the workflow for analysing Mutation prevalence in TB Drug Resistance.

An open-access dataset from the NIAID-NIH TB portal was used to analyze genomic mutations in patients with drug-resistant TB [25]. This dataset offers a comprehensive overview of gDST and whole-genome sequencing (WGS) analysis results for TB patients across 12 countries, covering the period from 2008 to August 2023. A key advantage of this dataset is its global coverage of bacterial strains, including recent isolates collected within the last 15 years, which increases the likelihood of capturing emerging strains. The dataset encompasses results from multiple TB diagnostic methods, ranging from traditional techniques such as microscopy and culture to advanced tests including MGIT Bactec, first- and second-line probe assays, and GeneXpert. Each record is associated with a specific patient case via a unique identifier, the “Condition ID.” The dataset contains 4,438 entries, comprising 1,373 drug-sensitive cases and 3,065 drug-resistant cases, which are further classified into categories such as RR-DR, Hr-DR, poly-DR, pre-XDR, MDR, non-XDR, and others. 1373 drug-sensitive mutations were excluded because the primary objective of this study was to perform multi-class classification among different drug-resistant types, rather than binary classification of resistance versus sensitivity. Including sensitive mutations would shift the modelling task toward a binary framework and could bias the model toward distinguishing sensitive versus resistant cases, rather than capturing mutation patterns specific to different resistance types. Moreover, drug-sensitive isolates generally lack resistance-associated mutations, which could introduce numerous non-informative features and dilute the model’s capacity to detect subtle mutation signatures among resistant classes. By restricting the analysis to drug-resistant isolates, the framework more effectively identifies mutation profiles specific to each resistance category. Patients received treatment regimens involving one to 16 drugs; some received only a single drug due to being in the initial treatment stage. The data document various mutations associated with resistance to both first- and second-line drugs, including rifampicin, isoniazid, ethambutol, pyrazinamide, streptomycin, amikacin, capreomycin, cycloserine, fluoroquinolones, kanamycin, bedaquiline, clofazimine, delamanid, ethionamide, levofloxacin, linezolid, moxifloxacin, ofloxacin, para-aminosalicylic acid, and aminoglycosides. Drug-specific resistance mutations occur at defined genomic loci. With continued drug exposure, mutations accumulate in the targeted gene or genes. For example, the first-line drug rifampicin targets only the *rpoB* gene, whereas isoniazid affects both the *inhA* and *KatG* genes (S2 Table). For this study the 3,065 drug-resistant patient details, including WGS analysis results and drug-resistant mutations associated with 21 drugs in the first- and second-line categories, were used to create the model. The mutation dataset had to be prepared for analysis. So, we did some cleaning. We removed terms like “NULL” and “–” from the data to ensure consistency. Then we looked at each piece of data and found the individual mutations. These mutations were listed as comma-separated strings, so we had to extract them. After that, we reformatted the mutations, each mutation into its feature. Subsequently, feature engineering was carried out by transforming these mutations into a binary format. Each mutation was represented as a separate feature with values indicating its presence (1) or absence (0) in each sample. Finally, feature exploration was conducted by calculating mutation frequencies across the dataset, enabling an understanding of the distribution and prevalence of different mutations. Features were aggregated based on resistance-associated mutations for 16 drugs based on the number of mutations reported associated to each drug, including rifampicin, isoniazid, ethambutol, pyrazinamide, streptomycin, amikacin, capreomycin, cycloserine, fluoroquinolones, kanamycin, bedaquiline, clofazimine, delamanid, ethionamide, levofloxacin, linezolid, moxifloxacin, and ofloxacin. The mutations are not available for all the 21 drugs some drugs have no mutation information if it is there also number of mutations is very low. Bedaquiline is one such drug we removed. The resulting matrix contains 654 features representing resistance-associated genes, such as *rpoB*, *rpoC*, *ahpC*, *katG*, *fabG1*, *inhA*, *embA*, *embB*, *pncA*, *gid*, *rpsL*, *rrs*, *eis*, *tyla*, *gyrA*, *gyrB*, *ald*, *alr*, and *ethA*. These loci encode determinants of resistance to tuberculosis drugs. The study workflow includes data acquisition from the NIAID-NIH TB portal, curation and construction of the binary mutation matrix, and subsequent computational analyses, as illustrated in Fig 1.

### 2.2. Machine learning model building and evaluation

All experiments were conducted on a Windows 11 system with 16 GB RAM, using Python 3.12.7. Key libraries included scikit-learn v1.3.1 for model development and evaluation, and SHAP v0.42.1 for interpretability analysis. The machine learning models were developed using a dataset split into training (80%) and test (20%) subsets. Classification algorithms applied included logistic regression, decision trees, random forests, AdaBoost, gradient boosting, support vector machines (SVMs), K-Nearest Neighbours (KNN), and Gaussian Naive Bayes. Model performance in predicting feature importance across all mutations was assessed. Hyperparameter tuning was performed on the training set (X_train, y_train) using GridSearchCV with stratified 10-fold cross-validation. The independent test set was reserved exclusively for final evaluation and was never used during model training or parameter optimization. For the analysis test set remained completely independent. The SHAPley method was used to visualise and explain the contribution of individual features to the model results. The application of these machine learning models aimed to enhance predictive accuracy, facilitate exploratory analysis, and implement explainable methods, thereby providing insights into factors influencing TB and drug-resistant mutations. Model evaluation employed classification metrics to determine how effectively mutation patterns predicted resistance types, including RR-TB, Hr-TB, MDR-TB, Pre-XDR-TB, XDR-TB and others. This methodology enabled the identification of mutation signatures most informative for distinguishing drug-resistant TB phenotypes and established a foundation for interpretable feature analysis using SHAP in subsequent steps. Top-ranked genomic variants were identified by filtering features with non-zero SHAP values, reflecting their predictive relevance in distinguishing TB drug resistance type. A Venn diagram was generated using Venny 2.0 (https://bioinfogp.cnb.csic.es/tools/venny/) to visualise the distribution of these informative mutations across different resistance categories, facilitating the identification of shared and specific mutations among each drug-resistant type RR, HR, MDR-TB, and Pre-XDR-TB.

### 2.3. Comparing Machine Learning Predictions with the WHO Catalogue for identifying high-confidence mutations

This study analyses the significant correlation between mutations identified by SHAP analysis and those listed in the WHO mutation catalogues, which serve as standardized references for TB mutations. Results were compared with the WHO catalogue for mutations from all resistance classes. High-confidence hits were identified, specifically those labeled as “Associated with resistance (AwR)”, “Associated with resistance interim (AwRI)”, and “Mutations with uncertain significance (Unsert.sig)”. Drug-resistant mutations were identified across all four resistance types: RR-TB, Hr-TB, MDR-TB, and Pre-XDR-TB. A table mapping the mutation categories used in this study to the corresponding WHO classification framework includes key parameters such as mutation frequency, positive predictive value (PPV), odds ratio (OR), and statistical significance (**S3 Table**). Four WHO mutation catalogues were used in this study: two global editions (2021 and 2023) and two Indian catalogues (2021 and 2024). All catalogues were retrieved and analyzed between 05 August 2025 and 17 November 2025 [26–29].

### 2.4. Predicting protein structural stability upon point mutations

The major set of mutations identified in the dataset are point mutations. The role of single-nucleotide polymorphisms (SNPs) in disease progression and drug resistance is well documented. Drugs that target specific proteins can influence protein structural stability by inducing mutations. Resistance-associated mutations may alter protein stability, thereby affecting the functional activity and regulation of biomolecules. Unfolding free energy serves as a primary indicator of protein stability. Variations in protein stability are quantified by comparing the free energy differences between mutant and wild-type proteins. The change in free energy (ΔΔG, measured in kcal/mole) is calculated by subtracting the free energy of the wild-type protein from that of the mutant. A ΔΔG greater than zero indicates increased stability after mutation, while a ΔΔG less than zero indicates decreased stability. ΔΔG indicates whether a change is stabilizing or destabilizing, the magnitude of the change is also important for biological interpretation. Small changes in ΔΔG are generally considered negligible and may not significantly affect protein stability, whereas larger magnitude changes are more likely to have functional consequences on protein structure and activity. Computational tools such as I-Mutant 2.0 and PredictSNP are employed to evaluate mutation stability. PredictSNP integrates multiple prediction algorithms and assigns a confidence score to each prediction, reflecting the consensus among the integrated tools [30,31]. This study analyses the stability of common mutations identified across all resistance classes.

## 3. Results

### 3.1. Prevalence of Mutation Types in DR TB from the whole dataset

**Fig 2:**
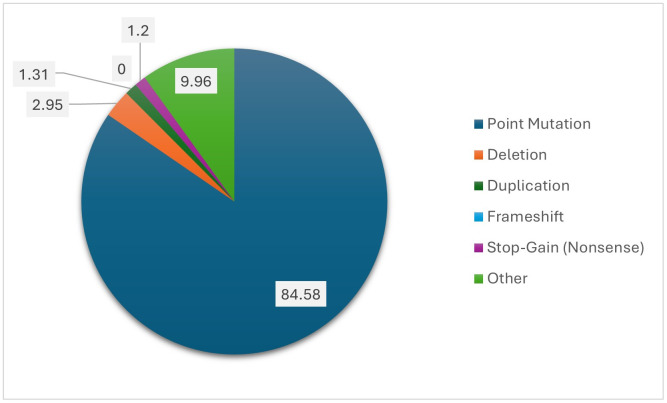
Percentage of different types of mutations available in the whole dataset.

A total of 3,065 participants with drug-resistant TB were included, each receiving a distinct regimen and displaying diverse drug resistance patterns. Mutations were classified into the following drug resistance categories: RR-TB, Hr-TB, MDR-TB, Pre-XDR-TB, and XDR-TB. The majority of resistance-conferring mutations were SNPs, accounting for 84.56% of cases. Deletions comprised 2.95%, while stop-gain mutations, which introduce a premature stop codon, represented 1.2%. No frameshift mutations were observed, and duplications accounted for 1.31%. Other mutation types constituted 9.96% (Fig 2).

### 3.2. Machine Learning Classifier and SHAP analysis Results

A total of 3,065 gDST and WGS results for drug-resistant TB were used to construct a ML model to predict the significance of genomic mutations associated with each drug resistance type. Mutation sets from the data frame were aggregated to create a feature matrix indicating the presence or absence of mutations across all drug targets. The target variable, drug resistance type, comprised categories such as RR-TB, HR-TB, MDR-TB, and Pre-XDR-TB. The dataset was partitioned into training (80%) and test (20%) sets for model development. Eight supervised ML algorithms were implemented, including Logistic Regression, Decision Tree, Random Forest, AdaBoost, Gradient Boosting, SVM, KNN, and Gaussian Naive Bayes. These models were trained on a binary mutation matrix to classify TB drug resistance phenotypes. To improve model performance and reliability, Random Forest hyperparameters were optimized via a grid search over n_estimators, max_depth, min_samples_split, and max_features. The optimized Random Forest model achieved 99% accuracy on both training and test sets, demonstrating robust predictive capability for resistance to drugs including rifampicin, isoniazid, ethambutol, pyrazinamide, streptomycin, amikacin, capreomycin, fluoroquinolones (levofloxacin, moxifloxacin, ofloxacin), aminoglycosides, cycloserine, kanamycin, and ethionamide. The classification report indicated high precision, recall, and F1-scores across resistance categories, except for the XDR-TB class, which showed comparatively lower performance (Precision: 0.87, Recall: 0.89, F1-score: 0.89) than the other resistance categories. This difference is primarily due to the limited number of XDR-TB samples in the dataset, leading to class imbalance. In contrast, classes such as MDR-TB and Hr-TB are better represented, allowing the model to learn more robust decision boundaries (Table 1).

**Table 1:** Classification Report for Random Forest Multi class TB drug resistance Classifier.

Drug Resistance Type	Precision	Recall	F1-score
HR-TB	1.00	0.99	0.99
MDR-TB	1.00	1.00	1.00
Pre-XDR-TB	0.99	1.00	1.00
RR-TB	0.93	0.96	0.95
XDR-TB	0.87	0.89	0.89
Others	1.00	0.98	0.98

**Fig 3:**
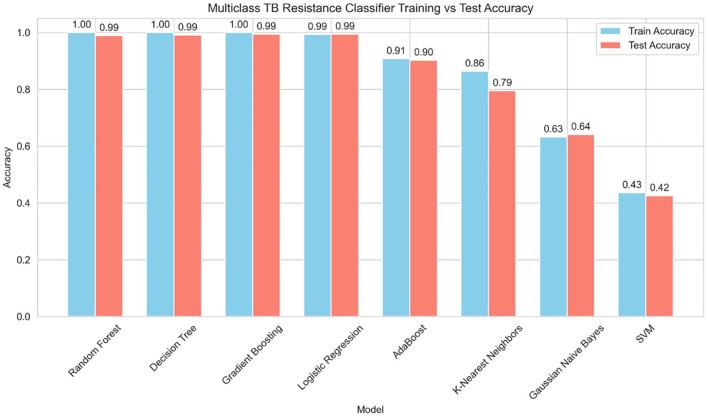
Performance of multiclass TB resistance classifier.

Random Forest was chosen as the base classifier for SHAP analysis because of its robust predictive performance and its capacity to manage high-dimensional mutation data (Fig 3).

The approach enabled quantification of feature contributions, thereby highlighting mutations with a consistent influence on resistance classification. SHAP values were calculated for each drug-resistant mutation in the test dataset. These values offer local interpretability by assigning a specific SHAP value to each feature or mutation in each sample, facilitating the identification of mutations contributing to classification of drug-resistance type in individual cases. The absolute SHAP values for each mutation were averaged across all samples, revealing mutations consistently associated with different type of drug resistance in the broader population. SHAP analysis was conducted on the test set using a Tree Explainer. The classification pipeline effectively identified TB drug resistance categories based on mutational data. SHAP-based feature interpretation provided both individual and global-level perspectives on the mutations most strongly influencing resistance type classification.

### 3.3. Comparative Interpretation of Resistance Mutations Using the WHO Catalogue

**Fig 4:**
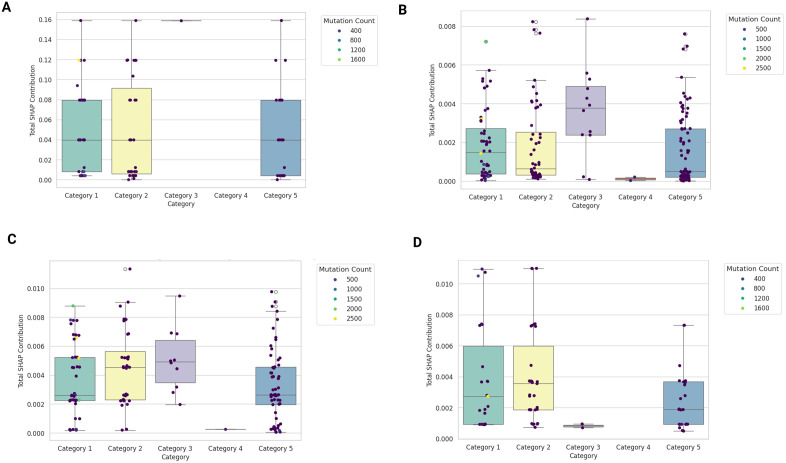
Box plots display SHAP contributions and the frequency of resistance mutations across five categories: Category 1, Category 2, Category 3, Category 4, and Category 5, not reported in the WHO catalogue. Panels show A) rifampicin-resistant TB (RR-TB); B) isoniazid-resistant TB (HR-TB); C) multidrug-resistant TB (MDR-TB); and D) pre-extensively drug-resistant TB (Pre-XDR TB).

**Fig 5:**
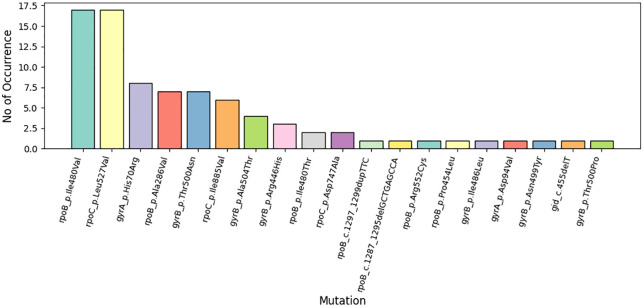
Bar chart depicting the distribution of mutations not reported in the WHO catalogues (Category 5) for rifampicin-resistant tuberculosis (RR-TB).

**Fig 6:**
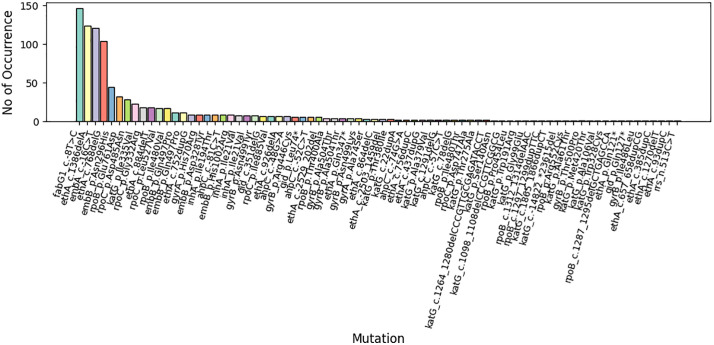
Bar chart depicting the distribution of mutations not reported in the WHO catalogues (Category 5) for HR-TB.

**Fig 7:**
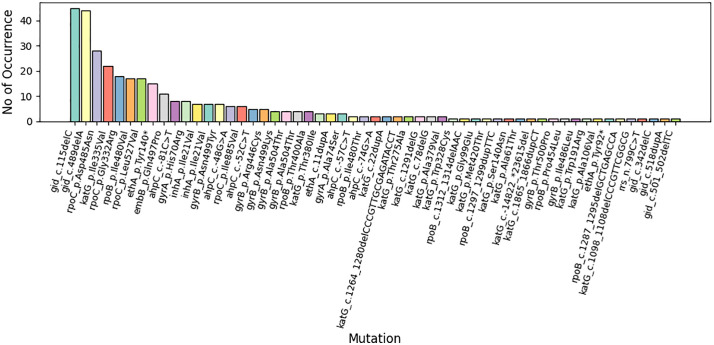
Bar chart depicting the distribution of mutations not reported in the WHO catalogues (Category 5) for MDR-TB.

**Fig 8:**
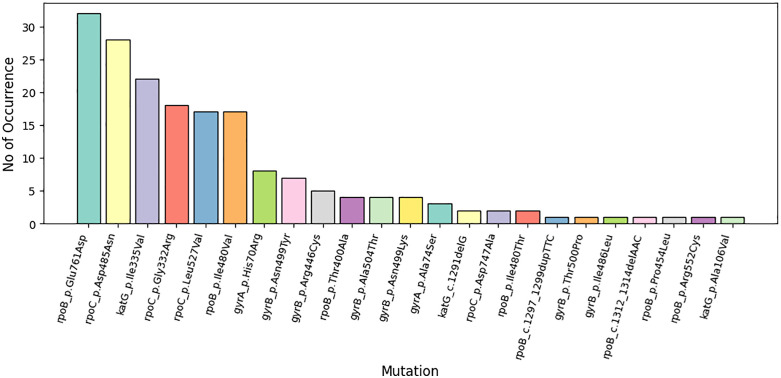
Bar chart depicting the distribution of mutations not reported in the WHO catalogues (Category 5) for Pre-XDR-TB.

The WHO mutation catalogue for *M. tb* classifies mutations into five groups, demonstrating a strong correlation between mutation significance and WHO gradings. This study compares four WHO catalogues: two global editions published in 2021 (first edition) and 2023 (second edition), and two catalogues derived from Indian datasets, specifically the Indian catalogue of *M. tb* mutations and associated resistance [26–29]. Results from the prediction of ML model were compared with the WHO catalogues to identify known resistance mutations and their corresponding confidence grading or resistance type for ML-predicted mutation lists from RR-TB, Hr-TB, MDR-TB, and Pre-XDR-TB. This comparison facilitates the identification of novel resistance mutations in each drug resistance type that are not reported in the catalogue. As outlined in the Methods section, resistance-associated mutations were identified by selecting features that maximally discriminate significant mutations in each resistance type, using the SHAP score. Mutations were segregated based on SHAP values and mutation counts. For each resistance type, results were compared with the WHO catalogue. According to the WHO confidence grading system, the first four categories represent mutations associated with resistance (S3 table). Category 1 includes mutations reported in the WHO “AwR” group, Category 2 includes mutations in the “AwRI” group, Category 3 corresponds to the “Uncert. Sig” group, Category 4 represents the “Not Associated with resistant interim (NotAwRI)” group, and Category 5 comprises “Mutations not reported in the WHO catalogue” (Fig 4). The accuracy of predicted resistance mutations for the 4-drug resistance type was evaluated by comparison with the WHO catalogue. Analysis of four resistance types identified a set of mutations that are not reported in WHO catalogues, each characterised by high SHAP values. The results suggest a potential association with resistance types; however, these mutations cannot be considered definitive novel markers, and their functional relevance requires further experimental and clinical validation. The frequency of these mutations was assessed across all resistance types. In RR-TB, several point mutations were identified in the *rpoB*, *rpoC*, *gyrA*, *gyrB*, and *gid* genes, none of which are currently documented in the four WHO mutation catalogue. Mutations such as *rpoC-I885V*, *rpoC-L527V*, *rpoB-I480V*, *rpoB-A286V*, *gyrAH70R*, *gyrB-A504T*, and *gyrB-R446H* were particularly frequent among the analysed isolates (Fig 5). Compared with RR-TB, Hr-TB exhibited a greater number of mutations not reported in any of the WHO catalogues. These mutations were distributed across several key genes, including *fabG1*, *ethA*, *embB*, *gyrA*, *gyrB*, *katG*, *rpoB*, and *rpoC*. The Hr-TB dataset also demonstrated a higher frequency of deletions and duplications relative to single-SNPs than observed in RR-TB. Among the identified SNPs, several mutations, including *rpoB*-E761D, *rpoC*-D485N, *rpoC*-G332R, *katG*-I335V, and *rpoB*-I480V, occurred at notably higher frequencies (Fig 6). Mutations not reported in the WHO catalogues for MDR-TB were identified across multiple genes, encompassing SNPs, deletions, and duplications. The specific mutations observed included *gid*-del115C, *gid*-del489A, *rpoC*-D485N, *katG*-I335V, *rpoC*-L527V, and *rpoC*-G332R. The presence of both deletions in gid and recurrent SNPs in *rpoC* and *katG* indicates a complex genetic basis for multidrug resistance (Fig 7). Pre-XDR-TB isolates exhibited fewer mutations not reported in the WHO catalogues than RR-TB, HR-TB, and MDR-TB isolates. Although the frequency of novel variants was lower, several unreported SNPs were identified in key resistance-associated genes, including *rpoB*-E761D, *rpoC*-D485N, *rpoC*-G332R, and *rpoB*-I480V, which appeared more frequently in the dataset. Some of these mutations exhibited high SHAP values, indicating their strong contribution to model predictions. These findings highlight potential candidate drug resistant mutations in *M. tb*, warranting further functional and clinical validation (Fig 8).

### 3.4. Common mutations segregated from all resistance types

**Fig 9:**
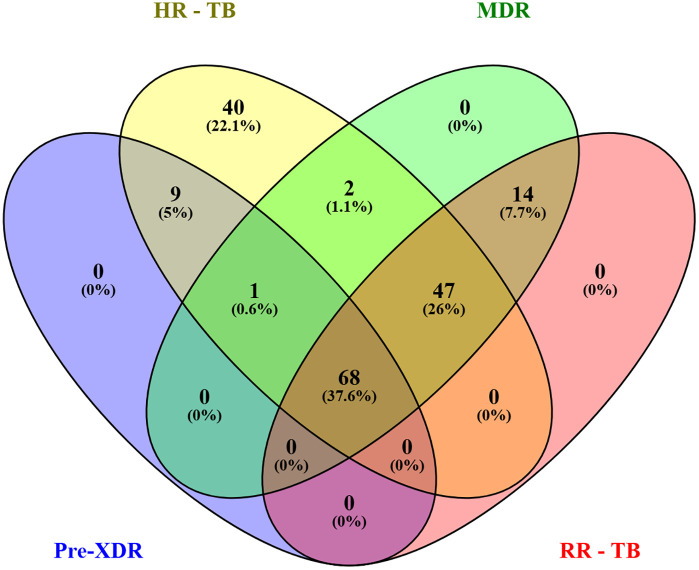
Venn diagram illustrating the common mutations identified among RR-TB, Hr-TB, MDR-TB, and Pre-XDR TB.

Feature importance, as determined by SHAP values for various types of resistance (RR-TB, Hr-TB, MDR-TB, and Pre-XDR TB), enabled the identification and analysis of 68 key mutations common to all four categories, as illustrated in a Venn diagram (Fig 9). These mutations predominantly occur in genes such as *rpoB, rpoC, gyrA, gyrB,* and *KatG*. Each mutation is classified by its frequency and prevalence across all candidate mutation lists. Prevalence is determined as the proportion of individuals in the population who carry the mutation (Table 2). The high prevalent mutations identified in the *rpoB* gene are S450L, S450F, S450W, H445D, H445N, H445Y, H445R, D435V, D435Y, L452P, and I480V. Notable mutations in the *rpoC* gene are D485N, G332R, and L527V. Prevalent mutations in *gyrA* include D94G, D94A, D94N, D94Y, and S91P. The *gyrB* gene exhibits mutations such as S447F, D461N, A504V, and D461H. The relevant mutation in *KatG* is I335V. The high prevalent canonical mutations identified are S450L, H445D, D435V, and H445Y in *rpoB*, and D94G in *gyrA*.

**Table 2:** The prevalence of each SNPs in the dataset is common to all drug-resistant types (RR-TB, HR-TB, MDR-TB, and Pre-XDR TB).

Gene Name	Mutation	No of occurrence in the dataset	Prevalence (%)
*rpoB*	S450L	1926	63.42
	S450F	132	4.35
	S450W	23	0.76
	S450G	2	0.07
	H445D	127	4.18
	H445Y	45	1.48
	H445N	42	1.38
	H445R	18	0.59
	D435V	88	2.9
	D435Y	61	2.01
	D435E	6	0.2
	D435A	3	0.1
	D435F	3	0.1
	L452P	37	1.22
	I480V	17	0.56
	I480T	2	0.07
	N437D	4	0.13
	T400A	4	0.1
	Q432K	3	0.1
	S428T	3	0.1
	L430R	3	0.1
	M434V	3	0.1
	M434I	1	0.03
	Q429H	3	0.1
	S441L	2	0.07
	T427S	1	0.03
	P454L	1	0.03
	N437S	1	0.03
	F170V	1	0.03
	L443W	1	0.03
*rpoC*	D485N	28	0.92
	G332R	18	0.59
	L527V	17	0.56
	F452S	1	0.03
*gyrA*	D94G	424	13.96
	D94A	132	4.35
	D94N	87	2.86
	D94Y	66	2.17
	D94H	16	0.53
	D94V	1	0.03
	S91P	101	3.33
	D89N	8	0.26
	H70R	8	0.26
	G88C	8	0.26
	G88A	5	0.16
	A74S	3	0.1
*gyrB*	S447F	25	0.82
	D461N	23	0.76
	A504V	23	0.76
	A504T	4	0.13
	D461H	13	0.43
	T500N	7	0.23
	T500P	1	0.03
	N499T	7	0.23
	N499Y	1	0.03
	R446C	5	0.16
	E501D	4	0.13
	R446H	3	0.1
	E501V	2	0.07
	I486L	1	0.03
*KatG*	I335V	22	0.72
	W300G	1	0.03
	A106V	1	0.03

### 3.5. Predicted functional impact and stability changes of mutations

I-Mutant 2.0 was used to calculate the ΔΔG value for predicting protein stability. Analysis of the predicted ΔΔG scores revealed that 17 out of 65 mutants exhibited increased stability, while the remaining mutants demonstrated decreased stability. For *rpoB* mutations, 9 out of 30 resulted in increased stability, whereas the others led to decreased stability. All mutations in *rpoC, gyrA, and katG* were associated with decreased protein stability. In contrast, 5 out of 14 *gyrB* mutations increased stability (Table 3).

**Table 3:** Presents the stability analysis results of single-nucleotide polymorphisms (SNPs) for common mutations identified in RR-TB, HR-TB, MDR-TB, and Pre-XDR-TB, as determined by I-Mutant 2.0. The table also indicates whether each mutation is reported in any of the four WHO catalogues, using the five-level confidence grading system including 4 categories, as well as mutations not reported in the catalogues (Category 5).

Gene Name	Mutation	Stability	ΔΔG Value (Kcal/Mol)	Reported in WHO catalogue
*rpoB*	A286V	Increase	0.93	Not Reported
	T427S	Increase	0.12	RRDR- AwRI
	Q429H	Decrease	−1.14	RRDR- AwRI
	L430R	Decrease	−0.64	RRDR- AwRI
	L430P	Decrease	−1.34	Borderline- AwRI
	Q432L	Increase	0.5	AwR
	Q432K	Decrease	−0.61	AwR
	Q432P	Decrease	−1.58	AwR
	M434V	Increase	0.4	RRDR- AwRI
	D435A	Increase	0.63	RRDR- AwRI
	D435Y	Increase	0.32	AwR
	D435G	Decrease	−0.54	RRDR- AwRI
	D435N	Decrease	−0.62	RRDR- AwRI
	N437S	Decrease	−0.75	RRDR- AwRI
	S441L	Decrease	−0.33	AwR
	S441Q	Decrease	−0.26	AwR
	L443W	Increase	0.48	RRDR- AwRI
	T444P	Decrease	−1.23	RRDR- AwRI
	H445D	Decrease	−0.15	AwR
	H445N	Decrease	−2.31	AwR
	S450L	Decrease	−0.48	AwR
	L452P	Increase	0.22	AwR
	P454R	Decrease	−0.63	Not Reported
	I480T	Decrease	−1.86	Not Reported
	I480V	Decrease	−0.51	Not Reported
	S493L	Decrease	−0.52	Not Reported
*rpoC*	G332R	Decrease	−1.11	Not Reported
	F452S	Decrease	−1.84	Not Reported
	D747A	Decrease	−1.73	Not Reported
	I885V	Decrease	−1.06	Not Reported
*KatG*	A61T	Decrease	−1.23	Not Reported
	G99E	Decrease	−0.41	Not Reported
	A109V	Decrease	−0.42	Uncert. Sig
	S315T	Increase	0.87	AwR
	S315N	Decrease	−0.18	AwR
	I335V	Decrease	−0.92	Not Reported
	T380I	Decrease	−0.26	Not Reported
	D419H	Decrease	−1.22	Uncert. Sig
	Q525P	Decrease	−1.64	Uncert. Sig
*gyrA*	D94Y	Decrease	−0.45	AwR
	D94A	Decrease	−0.5	AwR
	D94H	Decrease	−0.7	AwR
	D94G	Decrease	−1.51	AwR
*gyrB*	R446C	Decrease	−0.72	Not Reported
	R446H	Decrease	−0.31	Not Reported
	S447F	Decrease	−0.2	AwRI
	D461N	Decrease	−1.99	AwRI
	N499D	Increase	0.34	AwRI
	T500N	Decrease	−0.74	AwRI
	T500P	Decrease	−1.95	Not Reported
	E501V	Increase	0.9	AwRI
	A504V	Increase	0.12	AwRI

In addition to these predictions, PredictSNP was employed to assess the impact of mutations on function of the protein. PredictSNP integrates six leading prediction methods, offering a more accurate and robust alternative to individual tools such as I-Mutant 2.0. The suite of tools such as PredictSNP, MAPP, SIFT, Polyphen-1, Polyphen-2, and SNAP, determines whether protein mutations are deleterious or neutral based on predicted scores. The most common mutations, such as S450L, S450F, and H445D in *rpoB*, were associated with decreased stability in I-Mutant analyses, and all predictive SNP tools indicated deleterious effects (S4 Table).

### 3.6. Predicted Functional Impact and Stability Changes of Mutations Not reported in the WHO catalogue

A total of 40 SNPs were identified that have not been reported in any available version of the WHO mutation catalogue. These previously unreported SNPs were selected for detailed computational evaluation to assess their potential effects on protein structure and stability. Protein stability predictions for these SNPs were conducted using two web-based tools, I-Mutant 2.0 and PredictSNP, as described previously. The analysis reveals the potential destabilising or stabilising effects of the identified amino acid substitutions. These SNPs were found in seven genes: *rpoB*, *rpoC*, *gyrA*, *gyrB*, *katG*, *inhA*, and *embB*. The detailed results are presented in S5 Table. Mutations classified as deleterious by all SNP prediction algorithms and associated with decreased stability in I-Mutant results are listed in Table 4.

**Table 4:** Presents single-nucleotide polymorphisms (SNPs) not reported in all versions of the WHO mutation catalogues (category 5), which were analyzed to predict their effects on protein stability and function. I-Mutant 2.0 predicted that these mutations decrease protein stability, and all individual algorithms within Predict SNP, including MAPP, PhD-SNP, PolyPhen-1, PolyPhen-2, SIFT, and SNAP, classified the mutations as deleterious for the four analyzed proteins.

Gene name	Mutation position	I-Mutant 2 Result	ΔΔG (Kcal/mol)	Predict SNP Results						
				Predict SNP	MAPP	PhD-SNP	Polyphen −1	Polyphen −2	SIFT	SNAP
*rpoC*	L527V	Dec	−2.02	Del	Del	Del	Del	Del	Del	Del
*rpoB*	P454L	Dec	−0.45	Del	Del	Del	Del	Del	Del	Del
	I480T	Dec	−1.86	Del	Del	Del	Del	Del	Del	Del
*gyrA*	D94V	Dec	−0.38	Del	Del	Del	Del	Del	Del	Del
*KatG*	G99E	Dec	−0.41	Del	Del	Del	Del	Del	Del	Del
	W191R	Dec	−2.15	Del	Del	Del	Del	Del	Del	Del
	W328C	Dec	−2.26	Del	Del	Del	Del	Del	Del	Del
	T380I	Dec	−0.26	Del	Del	Del	Del	Del	Del	Del
	M420T	Dec	−0.8	Del	Del	Del	Del	Del	Del	Del

*Del – deleterious, *Dec – Decrease.

## 4. Discussion

According to the global tuberculosis report of the WHO published in 2024, an estimated 10.7–10.8 million people developed TB worldwide in 2023, with approximately 1.23 million deaths reported globally [1,32,33]. Drug-resistant TB is a major challenge despite the diagnosis and treatment of the disease. Especially, MDR-TB and XDR-TB pose extremely challenging effects in disease control. A major percentage of global TB incidence is present in low and middle-income countries. The transmission of drug-resistant strains hinders global TB control efforts. In this context, understanding the genomic basis of drug resistance, including mutation patterns and lineage-specific associations, helps enhance diagnosis and treatment approaches [34]. TB treatment involves a combination of first- and second-line drugs. Prolonged use of these regimens for 6–9 months can result in drug resistance due to mutations in the *M. tb* genome. These resistant strains subsequently spread within populations, complicating disease management and facilitating the emergence of MDR and XDR-TB [35]. pDST, gDST, and WGS are employed to identify drug-resistant strains and mutation sites. Early diagnosis of drug-resistant TB enables more effective treatment and recovery strategies. Mutations are specific to each anti-TB drug, as each targets distinct regions of the *M. tb* genome, leading to mutations in specific target genes. Mutations in rpoB (rifampicin resistance), katG, inhA, fabG1 (isoniazid resistance), embB (ethambutol resistance), and pncA (pyrazinamide resistance) are linked to first-line drugs. Mutations in gyrA and gyrB (fluoroquinolone resistance), rrs and eis (aminoglycoside resistance) are associated with second-line drugs, including fluoroquinolones (ofloxacin, moxifloxacin, levofloxacin) and injectable agents (amikacin, kanamycin, capreomycin). Additional genes such as ethA, inhA, alr, and atpE are linked to second-line drugs including ethionamide, cycloserine, clofazimine, and bedaquiline. This study analyzes genomic mutations in *M. tb* using a global dataset from 12 countries, with data obtained from the NIAID-NIH TB portal.

**Fig 10:**
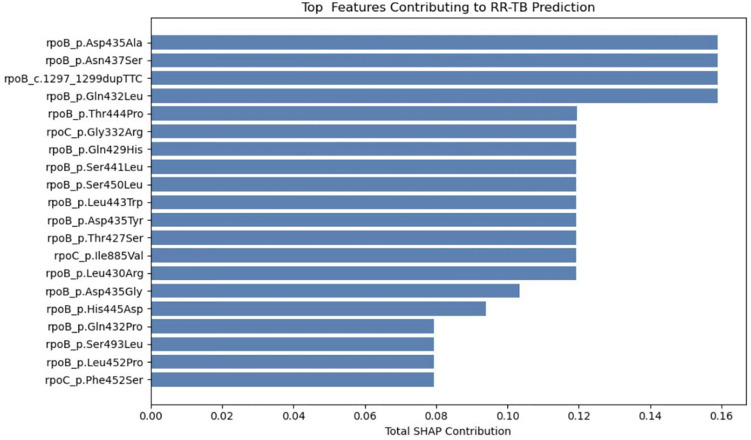
The bar graph displays significant mutations identified in rifampicin-resistant tuberculosis (RR-TB) alongside their corresponding SHAP values, which quantify the contribution of each mutation.

The decreasing cost of WGS has enhanced the availability of WGS data paired with pDST profiles for *M. tb*. These data are accessible through several public repositories, such as the Comprehensive Resistance Prediction for Tuberculosis (CRyPTIC) database [36], Relational Sequencing TB Data Platform (ReSeqTB) [37], Bacterial and Viral Bioinformatics Resource Center (BV-BRC), formerly the PATRIC database [38], Tuberculosis Drug Resistance Mutation Database (TBDReaMDB) [39], and Genome-wide *M. tb* Variation Database (GMTV) [40], which function as primary knowledge bases. Unlike these resources, the NIH-TB portal focuses exclusively on drug resistance mutations in global strains, which has led to its relative underutilization. In the present study, this dataset is employed for the first time to investigate drug-resistant mutations across various resistance types. The analysis enables the identification of novel mutations within each drug-resistant category that may serve as resistance-associated markers. Mutation data were processed using several ML algorithms, including Random Forest, Decision Tree, K-Nearest Neighbours, Gradient Boosting, AdaBoost, SVM, Logistic Regression, and Gaussian Naive Bayes. The Random Forest classifier was chosen for SHAP analysis due to its superior performance. Additionally, the study explores specific types of drug resistance and their associated mutations. For RR-TB, the most significant features associated with resistance are identified and displayed in a bar chart (Fig 10).

**Fig 11:**
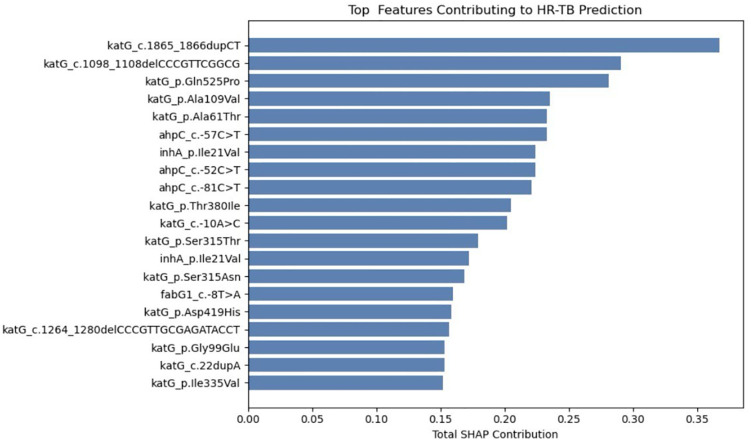
The bar graph shows significant mutations associated with isoniazid-resistant tuberculosis (HR-TB) and reports the SHAP values quantifying each mutation’s contribution.

A duplication at position 1299 (sequence TTC) and three missense mutations-D435A, N437S, and Q432L demonstrate the highest contribution to resistance, each with SHAP values of 0.16. All three missense mutations are located within the RRDR of the rpoB gene. Additionally, 10 other missense mutations, each with a SHAP value of 0.12, are frequently reported globally as rpoB mutations. Among these, S450L is recognised in the WHO mutation catalogue as associated with resistance and is highly prevalent in rifampicin-resistant cases, as reported in previous studies [41,42]. Two compensatory mutations in the *rpoC* gene, G332R and I885V, have also been reported. These mutations are less extensively studied and have primarily been identified in country-level investigations [43,44]. H445D and L452P, two mutations in the *rpoB* gene located within the RRDR, have SHAP values of 0.08. Additional mutations, including T444P, Q429H, S441L, L443W, T427S, and L430R (SHAP value 0.12), are listed in the WHO catalogue as either associated with resistance or classified as borderline. The D435Y mutation is specifically associated with resistance. The primary factor contributing to Hr-TB is mutations in the *KatG* gene. In contrast to RR-TB, Hr-TB exhibits additional mutation types, such as duplications and deletions, which contribute to isoniazid resistance beyond the commonly observed missense mutations. Mutations in *ahpC*, *inhA*, and f*abG1* also contribute to high-level resistance in tuberculosis (Fig 11).

**Fig 12:**
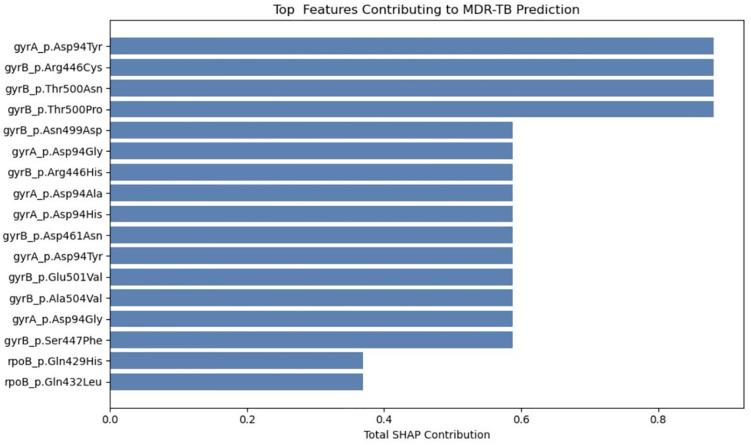
Primary genetic determinants contributing to multidrug-resistant tuberculosis (MDR-TB) and the corresponding SHAP values for each specific mutation.

**Fig 13:**
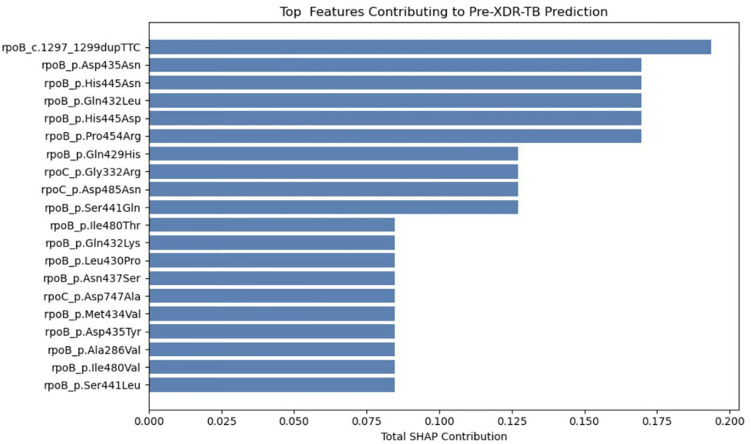
Mutations associated with Pre-XDR tuberculosis and their corresponding SHAP values.

The *KatG* S315T/N missense mutation is a well-documented variant in the WHO catalogue and is associated with drug resistance. These mutations represent the most common cause of isoniazid resistance in *M. tb*. The S315T mutation is highly prevalent and extensively studied in the context of HR-TB (SHAP Value: −0.17) [45–49]. Mutations in *gyrA* and *gyrB* are significant contributors to MDR-TB. Notably, *gyrA*-D94Y, *gyrB*-R446C, and T500N/P demonstrate the highest SHAP values, each exceeding 0.8 (Fig 12). Among these mutations, *gyrA*-D94Y is the most prevalent variant associated with MDR-TB, conferring resistance to fluoroquinolone (FQ) antibiotics. FQs, including Levofloxacin, Moxifloxacin, and Ofloxacin, are commonly used as second-line drugs for treating MDR-TB. The *gyrA*-D94Y mutation is classified as “Associated with resistance” according to the WHO mutation classification [50] and occurs within the quinolone-resistant determining regions (QRDRs). Mutations in *gyrA* and *gyrB* are the primary drivers of FQ resistance, which poses a significant challenge in MDR-TB management [51–53]. Specifically, the D94N, D94G, and D94Y substitutions in *gyrA* are strongly implicated in FQ resistance. In the Pre-XDR TB chart, rifampicin resistance mutations are the major contributors. The *rpoB*-H445N and P454R mutations are well-documented, frequently occurring variants, and are also categorised as “AwR” in the WHO catalogue (Fig 13).

**Fig 14:**
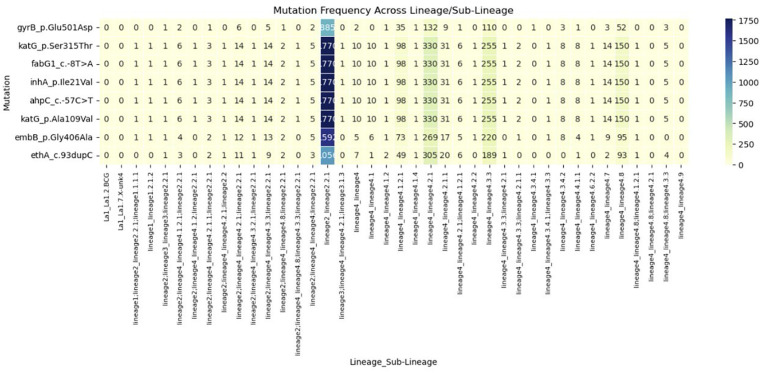
Mutation distribution on different lineages and sub-lineages associated with all drug-resistant types.

The heatmap displays the frequency of specific genetic mutations across different *M. tb* lineages and sub-lineages (Fig 14). Lineage 2.2.1 exhibits a high frequency of nearly all listed mutations, as indicated by dark blue cells with frequencies exceeding 1700. In contrast, many lineages at the far left and right of the heatmap display very low mutation counts, represented by light-yellow cells, indicating that these mutations are rare outside the 2.2.1 lineage cluster. The heatmap demonstrates that drug-resistance-related mutations are highly concentrated in the Beijing lineage (lineage 2.2.1) compared to other *M. tb* lineages. These results suggest that effective control of drug resistance requires focused attention on this sub-lineage in both treatment and surveillance strategies. Lineage 2.2.1 is a modern sub-lineage of the broader Beijing family (lineage 2) and has recently become globally prevalent, particularly in East Asian countries [54]. Another globally prevalent lineage is lineage 4, and the present results reflect the frequency of sub-lineages 4.2.1, 4.3.3, and 4.8. Previous studies have shown that these lineages are strongly associated with the development of MDR-TB and pre-XDR TB [55]. The current findings also indicate that *KatG*-S315T, *gyrB*-E501D, *inhA*-I21V, and *kat*GA109V mutations are associated with MDR and XDR-TB [56,57]. The relationship between lineage distribution and the existence of resistance-associated mutations was assessed using Fisher’s exact test. Lineage 2.2.1 (Beijing lineage) has statistically significant enrichment, which is closely linked to *katG* S315T, fabG1 c.-8T > A, *inhA* I21V, and *ahpC* c.-57C > T (Odds Ratio = 3.85, p < 3.1 x 10 ⁻ ²). *EmbB* G406A also demonstrated significant enrichment in Lineage 2.2.1 (Odds Ratio = 3.29, p ≈ 4.87 × 10 ⁻ ²). The Lineage 4 sub-lineages (e.g., lineage4.2.1 and lineage4.3.3) showed considerable enrichment, especially for variants such as *katG* S315T, fabG1 c.-8T > A, *inhA* I21V, and *ethA* c.93dupC, with statistically significant p-values (p < 0.001) and odds ratios. These results confirm that resistance-associated mutations are not uniformly distributed but show significant lineage-specific enrichment (S6 Table).

**Fig 15:**
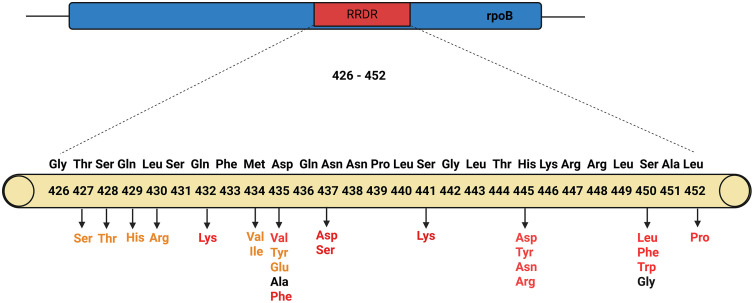
Significant rifampicin resistance mutations identified in the present analysis are located within the rifampicin resistance-determining region (RRDR) of the *rpoB* gene.

The frequency of each mutation was assessed. The most prevalent mutations in *rpoB* were S450L (63.4%), S450F (4.35%), and H445D (4.18%). These three mutations are significantly associated with rifampicin resistance and are listed in the WHO catalogue as “AwR” (Table 4). All *rpoB* mutations identified in this study are located within the RRDR, an 81-base pair segment of the *rpoB* gene spanning codons 507–533, where most mutations conferring rifampicin resistance are found (Fig 15).

This region encodes a segment of the RNA polymerase enzyme, which is the primary target of rifampicin. Mutations within this region disrupt rifampicin binding and confer rifampicin resistance. In *M. tb*, although such mutations can reduce bacterial fitness, compensatory mutations in the *rpoA* and *rpoC* genes may restore fitness in resistant strains, as reported in previous studies [58–61]. In the present analysis, *rpoC* mutations D485N (0.92%) and G332R (0.59%) were identified; neither mutation is reported in any of the WHO catalogues (Table 4). FQs are the primary therapeutic agents for DR-TB. Resistance to FQs in tuberculosis is associated with mutations in the quinolone resistance-determining region (QRDR) of the *gyrA* and *gyrB* genes, which encode type II DNA topoisomerase. Mutations in *gyrA* contain high-level resistance, whereas those in gyrB are associated with low-level resistance. The five most prevalent *gyrA* mutations are D94G (13.9%), D94A (4.35%), D94N (2.86%), D94Y (2.17%), and S91P (3.33%). D94G, D94N, D94Y, D94A, and S91P are common mutations associated with FQ resistance, as reported in several previous studies, and all are located within the QRDR [62–68]. The three FQs used for the treatment of MDR-TB – levofloxacin, moxifloxacin, and ofloxacin are associated with the development of drug resistance. The D94G, D94N, D94Y, and D94A mutations identified in this study are also reported in the WHO mutation catalogue as being associated with resistance and resistance intermediates. In *gyrB*, the highest mutation frequencies observed were S447F (0.82%), D461N (0.76%), A504V (0.43%), and D461H (0.43%) [69]. Both D461N and E501D are reported in the WHO mutation catalogue is associated with resistance, with E501D being the only mutation, other than D461N, listed in this category. The *KatG* mutation in TB refers to a specific change in the *katG* associated with resistance to the antibiotic isoniazid. Studies have reported varying prevalence of the *KatG* mutation. For *KatG*, I335V (0.72%) was the most prevalent mutation, and a total of three mutations were reported as significant in this study. None of these three mutations is reported in the WHO catalogues’ resistance categories [70].

Mutations associated with four resistance types RR-TB, Hr-TB, MDR-TB, and Pre-XDR TB were compared with the WHO mutation catalogues. These mutations comprise SNPs, deletions, and duplications. A subset of SNPs was identified that were not reported in any version of the catalogues. These mutations were analysed using I-Mutant 2, a tool that predicts SNPs likely to decrease protein stability and be del. In *rpoC*, the mutations G332R and L527V were identified. The *rpoC*-G332R mutation has been described in previous studies as a non-synonymous mutation, but it remains rare globally (<0.05%) and is exclusively observed in RR-TB cases [71]. The *rpoC* mutations are compensatory and can restore fitness lost due to primary drug resistance mutations in *rpoB* [44,72,73]. L527V has not been previously reported and was first identified in this analysis; it may serve as a marker mutation. In *rpoB*, I480 is an amino acid position located just outside the 81-base pair RRDR region [74]. Previous studies have reported the substitution I480V at this position [75]. In the present dataset, a novel variant, I480T, was identified, while D94A is the most frequently occurring marker of FQ resistance [76,62]. These mutations, located in the QRDR within the bacterial genome, are also reported in the WHO catalogues. D94V, the mutation reported in this dataset, is less frequent (1–2%) and has been previously described [76–78]. In *KatG*, associated with isoniazid resistance, the mutations G99E, A106V, W191R, W328C, T380I, and M420T are not reported in the WHO catalogues; all are predicted to be deleterious. Except for M420T, the remaining mutations have been previously reported in a limited number of studies, although they have been less thoroughly explored and analysed [79–83]. Employing computational tools to assess the structural stability and pathogenicity of mutated genes or proteins is essential for understanding the extent of drug resistance conferred by specific genomic changes. This study utilised PredictSNP2 and I-Mutant-2 to assess the structural stability and mutation impact of specific proteins. The results are discussed above, and each tool serves a distinct purpose. I-Mutant-2 predicts the effect of SNPs on protein stability classification and estimates ΔΔG values via regression. PredictSNP is a classifier that distinguishes between deleterious and neutral SNPs, utilising various algorithms for this classification. SIFT, Polyphen, and PredictSNP have demonstrated superior classification performance compared to other algorithms.

The developed ML model predicts drug resistance types and identifies drug resistance-related mutations, including SNPs and structural variants such as deletions and duplications. Previous studies have investigated the use of ML models for predicting *M. tb* resistance phenotypes, asserting that their algorithms incorporate explainable artificial intelligence components and have identified the most significant variants within their datasets. In the present study, drug resistance is defined as combinatorial resistance profiles rather than outcomes for individual drugs. This approach enables the model to learn mutation patterns that co-occur across multiple drugs, which is essential for identifying structural variants, such as duplications and deletions that influence multiple resistance pathways. Predicting mutations associated with each drug-resistant type is directly relevant to patient treatment strategies and clinical outcomes. The ability to accurately predict resistance-associated mutations for each drug-resistant type also facilitates the identification of co-occurring mutations, rather than limiting analysis to mutations specific to individual drugs. Tuberculosis patients often receive multiple drugs over a period of six to nine months. Focusing exclusively on drug resistance mutations for individual drugs may not adequately capture the significance of co-occurring mutations that contribute to disease progression and prevalence. This framework advances understanding of the genetic basis of AMR and quantifies the critical SNPs that inform model decisions. Such improvements may enhance the interpretability of ML algorithms and support more informed clinical decision-making.

## 5. Limitations of the study

This study has several limitations. First, our dataset lacked information on certain drugs, such as bedaquiline, delamanid, linezolid, para-aminosalicylic acid, and clofazimine. Additionally, there was insufficient data on XDR-TB cases. XDR-TB represents the most severe form of drug resistance, involving resistance to both first, second-line drugs, and at least one fluoroquinolone. Due to the limited number of XDR-TB cases in our dataset, we were unable to include XDR-TB as a classifier in our analysis. Instead, we included pre-XDR cases, which are more resistant category than MDR-TB but less resistant than XDR-TB, as one of the classes for ML model development and optimization. Due to limited access to independent datasets with comparable genomic and phenotypic annotations, external validation was not performed.

## 6. Conclusion

This study developed a ML framework to investigate drug resistance mutations and the significance of specific mutations in various types of resistance, including RR-TB, HR-TB, MDR-TB, and pre-XDR-TB. Unlike previous research, this work emphasizes the distinct characteristics of each resistance type. The WHO mutation catalogue serves as the standard reference resistance associated mutations from global dataset. The analysis encompasses all mutation types, such as deletions, duplications, indel/structural variants, promoter/regulatory, non-sense (loss of function), and SNPs. The dataset demonstrates a substantial proportion of SNPs, highlighting their relevance to drug resistance and disease progression. Consequently, the primary focus is on SNPs identified across the four drug-resistant categories, with an emphasis on analysing their structural stability and pathogenicity. A set of candidate mutations were identified for each drug resistance type may be valid once could confirm once the experimental validation. Other than SNPs mutations from other categories were also show specific relevance. The results shows that the proposed model exhibits superior predictive capacity. The integration of ML with an enhanced model offers potential for addressing drug resistance in tuberculosis research and therapeutic interventions. Furthermore, an improved ML framework, supported by experimental validation, can advance the understanding of drug resistance patterns and inform future clinical decision-making.

## Supporting information

S1 Table Key definitions in drug resistant TB treatment according the WHO guidelines 2021, 2023. (DOCX)

S2 Table Various drugs are associated with specific target genes and distinct mutation types identified within the dataset. (DOCX)

S3 Table Classification of mutations based on WHO catalogue criteria, Category 1–4, and mutations not reported in the catalogues. (DOCX)

S4 Table Stability analysis results of SNPs for common mutations from RR-TB, HR-TB, MDR-TB, and Pre-XDR-TB using Predict SNP. (XLSX)

S5 Table Stability analysis results of 40 SNPs were identified that have not been reported in any of the available versions of the WHO mutation catalogue using I-mutant 2.0 and predict SNP. (XLSX)

S6 Table Results of Fisher’s exact test assessing the association between lineage distribution and the presence of resistance-associated mutations. (XLSX)

## Abbreviations

AwR: Associated with resistance
AwRI: Associated with resistance-interim
Uncert. Sig: Uncertain significance
NotAwR: Not Associated with resistance
RR-TB: Rifampicin Resistant TB
HR-TB: Isoniazid resistant TB
MDR-TB: Multidrug resistant TB
pre-XDR-TB: Pre-extensively Drug-Resistant TB
SHAP: SHapley Additive exPlanations
WHO: World health organisation
M. tb: Mycobacterium tuberculosis
FQ: Fluoroquinolone
QRDR: Quinolone resistance-determining region
AMR: Antimicrobial resistance
ML: Machine learning
RRDR: Rifampicin-resistant-determining region
gDST: Genotypic drug-susceptibility test
NIAID: National Institute of Allergy and Infectious Diseases
NIH: National institute of health
DR-TB: Drug-resistant tuberculosis
SNPs: single-nucleotide polymorphisms
