# Supplementary material for: Machine learning-based analysis of drug resistance mutations in Mycobacterium tuberculosis
Source: PLoS One. 2026 Jul 10;21(7):e0352863. doi: 10.1371/journal.pone.0352863 (PMC13354099; doi:10.1371/journal.pone.0352863)
Supplement: S1 Table — (DOCX) [file pone.0352863.s001.docx]

**S1 Table**: Key definitions in drug resistant TB treatment according the WHO guidelines 2021, 2023

| **Terms associated with drug resistance** | **Definition of the term** |
| --- | --- |
| Drug Resistant TB (DR-TB) | TB disease caused by a strain of *M. tb* complex that is resistant to any TB medicines |
| Drug susceptibility testing (DST) | In vitro testing using either molecular or genotypic techniques to detect resistance-conferring mutations, or phenotypic methods to determine susceptibility to a medicine |
| Rifampicin-resistant TB (RR-TB) | TB disease caused by a strain of *M. tb* complex that is resistant to rifampicin |
| Rifampicin-susceptible, isoniazid-resistant TB (Hr-TB) | TB disease caused by a strain of  *M. tb* complex that is resistant to isoniazid but susceptible to rifampicin |
| Multidrug-resistant TB (MDR-TB) | TB disease caused by a strain of *M. tb* complex that is resistant to rifampicin and isoniazid |
| Pre-extensively drug-resistant TB (pre-XDR-TB) | TB disease caused by a strain of *M. tb* complex that is resistant to rifampicin (and may also be resistant to isoniazid), and that is also resistant to at least one fluoroquinolone (either levofloxacin or moxifloxacin) |
| Extensively drug-resistant TB (XDR-TB) | TB disease caused by a strain of *M. tb* complex that is resistant to rifampicin (and may also be resistant to isoniazid), and that is also resistant to at least one fluoroquinolone (levofloxacin or moxifloxacin) and to at least one other “Group A” drug (bedaquiline or linezolid) |
