# Supplementary material for: Machine learning-based analysis of drug resistance mutations in Mycobacterium tuberculosis
Source: PLoS One. 2026 Jul 10;21(7):e0352863. doi: 10.1371/journal.pone.0352863 (PMC13354099; doi:10.1371/journal.pone.0352863)
Supplement: S2 Table — (DOCX) [file pone.0352863.s002.docx]

**S2 Table:** Various drugs are associated with specific target genes and distinct mutation types identified within the dataset

| **Drug** | **Drug targeting loci** | **Types of mutations** |
| --- | --- | --- |
| Rifampicin | *rpoB and rpoC* | SNPs, point mutations duplicates, and deletions |
| Isoniazid | *ahpC, katG, fabG1, and inhA* | SNPs, point mutations duplicates, and deletions |
| Ethambutol | *embA and embB* | SNPs and point mutations |
| Pyrazinamide | *pncA* | SNPs, point mutations duplicates, insertion and deletions |
| Streptomycin | *giD, rpsL, and rrs* | point mutations duplicates, and deletions |
| Amikacin | *rrs and eis* | Point mutation |
| Capreomycin | *rrs and tyla* | point mutations duplicates, and deletions |
| Fluoroquinolone | *gyrA and gyrB* | SNPs and point mutations |
| Levofloxacin | *gyrA and gyrB* | SNPs and point mutations |
| Moxifloxacin | *gyrA and gyrB* | SNPs and point mutations |
| Ofloxacin | *gyrA and gyrB* | SNPs and point mutations |
| Aminoglycosides | *rrs* | Point mutations |
| Cycloserine | *ald and alr* | point mutations duplicates, and deletions |
| Kanamycin | *eis and rrs* | point mutations and deletions |
| Ethionamide | *ethA, inhA, and fabG1* | point mutations duplicates, and deletions |
