# Supplementary material for: Machine learning-based analysis of drug resistance mutations in Mycobacterium tuberculosis
Source: PLoS One. 2026 Jul 10;21(7):e0352863. doi: 10.1371/journal.pone.0352863 (PMC13354099; doi:10.1371/journal.pone.0352863)
Supplement: S3 Table — (DOCX) [file pone.0352863.s003.docx]

**S3 Table:** Classification of mutations based on WHO catalogue criteria: Associated with resistance, Associated with resistance (interim), Uncertain significance, Not associated with resistance (interim), and Not associated with resistance

| **Categories** | **WHO Classification** | **Description** | **Criteria Summary** |
| --- | --- | --- | --- |
| **Category 1** | Associated with Resistance | High-confidence resistance associated mutations (Marker of resistance) | Mutations supported by sufficient sample size, high positive predictive value (PPV), odds ratio (OR > 1), and statistically significant association with resistance |
| **Category 2** | Associated with Resistant Interim | Moderate confidence resistance associated mutations (Likely resistance) | Mutations meeting relaxed criteria (e.g., ≥2 resistant isolates and PPV ≥ 50%) but lacking full statistical support |
| **Category 3** | Uncertain Significance | Insufficient evidence for classification (Uncertain significance) | Mutations that do not meet criteria for resistance or non-resistance categories |
| **Category 4** | Not associated with resistance-interim | Likely non-resistance associated mutations (Likely not resistance) | Mutations with low PPV and confidence intervals suggesting lack of association with resistance |
| **Category 5** | Not Associated with resistance | Neutral Mutations (Not a resistant marker) | Mutations identified as neutral or excluded based on lack of association with resistance |
